# Do non-communicable diseases such as hypertension and diabetes associate with primary open-angle glaucoma? Insights from a case–control study in Nepal

**DOI:** 10.3402/gha.v6i0.22636

**Published:** 2013-11-04

**Authors:** Suraj Shakya-Vaidya, Umesh Raj Aryal, Madan Upadhyay, Alexandra Krettek

**Affiliations:** 1 Department of Ophthalmology, Nepal Medical College Teaching Hospital, Kathmandu, Nepal; 2 Nordic School of Public Health NHV, Gothenburg, Sweden; 3 Department of Community Medicine, Kathmandu Medical College, Kathmandu, Nepal; 4 B.P. Eye Foundation, Kathmandu, Nepal; 5 Department of Internal Medicine and Clinical Nutrition, Institute of Medicine, Sahlgrenska Academy at University of Gothenburg, Gothenburg, Sweden

**Keywords:** non-communicable disease (NCD), hypertension, diabetes mellitus, blindness, primary open-angle glaucoma (POAG)

## Abstract

**Background:**

Non-communicable diseases (NCDs) such as hypertension and diabetes are rapidly emerging public health problems worldwide, and they associate with primary open-angle glaucoma (POAG). POAG is the most common cause of irreversible blindness. The most effective ways to prevent glaucoma blindness involve identifying high-risk populations and conducting routine screening for early case detection. This study investigated whether POAG associates with hypertension and diabetes in a Nepalese population.

**Methods:**

To explore the history of systemic illness, our hospital-based case–control study used non-random consecutive sampling in the general eye clinics in three hospitals across Nepal to enroll patients newly diagnosed with POAG and controls without POAG. The study protocol included history taking, ocular examination, and interviews with 173 POAG cases and 510 controls. Data analysis comprised descriptive and inferential statistics. Descriptive statistics computed the percentage, mean, and standard deviation (SD); inferential statistics used McNemar's test to measure associations between diseases.

**Results:**

POAG affected males more frequently than females. The odds of members of the Gurung ethnic group having POAG were 2.05 times higher than for other ethnic groups. Hypertension and diabetes were strongly associated with POAG. The overall odds of POAG increased 2.72-fold among hypertensive and 3.50-fold among diabetic patients.

**Conclusion:**

POAG associates significantly with hypertension and diabetes in Nepal. Thus, periodic glaucoma screening for hypertension and diabetes patients in addition to opportunistic screening at eye clinics may aid in detecting more POAG cases at an early stage and hence in reducing avoidable blindness.

Non-communicable diseases (NCDs) are public health challenges worldwide due to their rising prevalence and impact on quality of life from complications that lead to morbidity, mortality, and disability (1, 2). In 2000, the global prevalence of hypertension was 26.4% in adults, and it likely will reach 29.2% by 2025 (1). For diabetes, global prevalence was 8.3% in 2012 (3), and projections suggest that in low- and high-income countries prevalence will reach 69 and 20%, respectively, by 2030 (2). Currently, more than 70.3 million people in the Southeast Asia region (SEAR) have diabetes; by 2030, this will rise to 120.9 million (4). Economic transition, rapid urbanization and changing lifestyles, tobacco use, excessive alcohol consumption, and insufficient physical activity are the major risk factors for NCDs, the effects of which are seen increasingly in low- and middle-income countries (5).

In Nepal, hypertension prevalence in adults is 33.9% (6). Interestingly, the prevalence has tripled – from 6 to 18% – in rural Nepal during the last 25 years (7). According to studies conducted in different communities, diabetes prevalence in Nepal varies from 6.3 to 8.5% (7, 8).

Complications that accompany hypertension and diabetes affect various body organs, including the eye (5, 9). Diabetes and hypertension are also risk factors for developing the blinding eye condition primary open-angle glaucoma (POAG) (10, 11). However, to date there is a paucity of data from Nepal, and either positive or no associations are reported between these diseases (12, 13).

The International Agency for Prevention of Blindness (IAPB) listed glaucoma as the third largest cause of overall blindness in its 2010 report (14), and POAG is the most common type of glaucoma both worldwide (15) and in Nepal (16, 17). POAG is often called ‘a silent sight killer’ because of its slow and progressive nature, which leads to blindness without alarming symptoms (18). The most effective way to overcome glaucoma blindness is early detection and timely treatment before irreversible blindness occurs (19).

Vision 2020: The Right to Sight initiative (14) has identified a number of targets to prevent glaucoma blindness, which include glaucoma diagnosis by routine case detection rather than population-based screening, integrating glaucoma care into existing eye care initiatives, and conducting glaucoma research. Thus, the most important step toward preventing glaucoma blindness is to identify high-risk individuals attending healthcare centers to initiate routine case detection and direct them to health awareness and screening programs. Today in Nepal, opportunistic glaucoma screening is carried out in eye departments, eye hospitals, mobile cataract-screening camps, and research surveys (16, 20). The horizon of case detection for glaucoma needs to be widened by identifying all possible high-risk individuals attending healthcare centers.

If the association between diabetes, hypertension, and POAG holds true for the Nepalese population as well, future screening of patients who attend hospitals for routine follow-up of hypertension and diabetes and for eye check-up for retinopathies would be critical. To date, there are no reports from Nepal about this association, and conclusions vary widely from other parts of the world regarding the association of diabetes and hypertension with POAG (12, 13, 21). Therefore, we designed this study to determine whether hypertension and diabetes are associated with POAG in various ethnic groups of Nepal.

## Methods

### Study design and study sites

We conducted a hospital-based case–control study in three Nepalese hospitals between February 2010 and May 2011. To achieve a representative sample in terms of geography (i.e. the plains and mid-hills regions), major ethnic groups, and availability of the full range of diagnostic facilities required for glaucoma screening, we purposely selected Nepal Medical College, a teaching hospital in Kathmandu; Himalaya Eye Hospital in Pokhara; and Geta Eye Hospital in Dhangadi.

### Sampling techniques and sample size

We used a non-random consecutive sampling technique to enroll newly diagnosed POAG patients from the general eye clinics of each hospital. Published data on the prevalence of diabetes and hypertension (22–24) were considered to calculate the sample size. However, we used the proportion of hypertension in control groups, which was 0.12, with an odds ratio (OR) of 2.4, and we assumed that correlation between cases and controls was 0.225 (25) as this allowed us to obtain a larger sample size. We determined the minimum sample size in which to detect an OR similar to 2.4 as 168 cases and 504 controls (with three age-, gender-, and ethnicity-matched controls per case), with power of 90% at a 95% confidence interval (CI).

### Study population

Due to Nepal's diverse ethnicity and caste system, we sought to include individuals from various major ethnic groups (e.g. Newar, Brahmin, Gurung, and Tharu) living in distinct pockets of three developmental regions where the hospitals are located.

### Inclusion and exclusion criteria

We included Nepalese belonging to major ethnic groups. To achieve good cooperation during the special eye test for glaucoma diagnosis, we excluded individuals younger than 15 years of age. We also excluded individuals with secondary glaucoma, narrow angles, ocular pathologies that obscure the view of the optic nerve head, and pathologies that could alter intraocular pressure (e.g. uveitis and high refractive errors>5 dioptre).

### Ocular history and examinations

After obtaining an ocular history from all study participants, focusing on presenting symptoms, past ocular illness, and a history of use of any medications, we administered a visual acuity test, retinoscopy, and refraction, followed by anterior and posterior segment examination with a slit lamp and 90 D lens. We used a Goldmann Three Mirror Lens for gonioscopy and measured intraocular pressure with a Goldmann Applanation Tonometer (both manufactured by Haag-Streit, Koeniz, Switzerland). We tested the visual field using the full-threshold 24-2 program in a Humphrey Field Analyzer (Carl Zeiss, Oberkochen, Germany).

Next, we recorded participants’ vision as recommended by the 2002 Resolution of the International Council of Ophthalmology (26). To diagnose POAG, we followed the Asia Pacific Glaucoma Guidelines (27), based on the presence of an open anterior chamber angle with signs of glaucomatous optic neuropathy and a corresponding glaucomatous visual field defect with normal or high intraocular pressure.

### History taking for hypertension and diabetes

We interviewed all participants to determine any history of diabetes and hypertension. Both interviewer and participant were blinded. Participants had no access to the results of any examination or diagnosis until they had completed the diabetes and hypertension interview. The blinded interviewer received folded pages, secured with a sticker, of the clinical findings and diagnosis. A history of hypertension and diabetes was considered only if the individual provided a history of illness and was taking medication, as evidenced by a prescription. Participants who could not provide a prescription were asked to return the next day with all of their prescriptions.

### Data analysis

We entered the collected data into Microsoft Excel 2007 and performed statistical analysis using SPSS Statistics 17 (SPSS Inc., Chicago, IL, USA) and Stata10 software. We used both descriptive and inferential statistics for data analysis. In descriptive statistics, we computed the percentage, mean, and standard deviation (SD) to describe demographic characteristics and clinical variables.

In inferential statistics, we used McNemar's test to measure the association between POAG, hypertension, and diabetes. Data were expressed in a fourfold table containing concordant and discordant pairs. We defined the case–control pair as concordant when both or neither member of the pair had been exposed to hypertension or diabetes. A discordant pair showed mixed exposure between cases and controls. Finally, we computed the OR for discordant pairs (95% CI) and gender and caste groups within cases (95% CI) and set the *p* value (5% level of significance).

### Ethical considerations

The Nepal Health Research Council (ref no. 177/2066-05-10) and all participating hospitals approved this study. All study participants were informed about the study and its purpose. We also gave participants a detailed explanation of the examination procedure. Participants gave verbal informed consent and were informed that they could withdraw from the study at any time. POAG patients were treated with either anti-glaucoma medication or filtering surgery. Participants who could not afford treatment were treated at no cost at participating hospitals.

## Results

### Demographic profile

Table 1 describes the demographic characteristics of all individuals attending hospital during the study period, including the study participants. Among 4,463 individuals aged 15 years and older who were attending the general eye clinic, 183 (4.1%) were diagnosed with POAG for the first time. Among those, 173 fulfilled the study's enrollment criteria. Thereafter, 510 participants attending hospital who did not have POAG (i.e. they were diagnosed either as normal with no ocular disorders or as having just a refractive error) were enrolled as controls.

**Table 1:** Demographic characteristics of cases and controls

Variable	Total adults attending hospital	Total cases (%) (POAG)	Total controls (%) (without POAG)	Odds ratio (95% CI) for cases only

		*N*=173	*N*=510	
Gender				
Female	2,245	50 (2.22)	145 (6.45)	Reference
Male	2,218	123 (5.50)	365 (16.40)	2.58 (1.82–3.65)
Age group (years)				
15–35	790	13 (1.65)	39 (4.93)	Reference
36–55	1,585	55 (3.47)	168 (10.6)	2.15(1.13–4.15)
56–75	1,380	82(6.02)	239 (17.6)	3.90 (2.10–7.39)
>75	728	23(3.15)	64 (8.79)	1.90 (0.92–4.04)
Mean±SD		58.90±14.72	58.54±14.68	
Ethnic group				
Brahmin	1,380	48 (3.47)	142 (10.29)	Reference
Newar	1,538	54 (3.51) *p=0.76*	159 (10.33)	1.16 (0.76–1.79)
Gurung	689	43 (6.24) *p=0.002*	126 (18.28)	2.05 (1.30–3.24)
Tharu	859	28 (3.25) *p=0.61*	83 (9.66)	1.07 (0.69–1.79)

CI: confidence interval; POAG: primary open-angle glaucoma; SD: standard deviation.

The sex ratio of the patients was 2.46 males to 1 female; the mean age was 58.9±14.7 years. Nearly 80% of the cases attending the hospitals in this study were between the ages of 36 and 75 years, 7.5% were younger than 36 years, and 13.3% were older than 75 years. Among all participants, 31.2% were Newar, followed by Brahmin (27.7%), Gurung (24.9%), and Tharu (16.2%).

Based on the total number of individuals in each ethnic group attending the hospital, POAG was higher among Gurung (6.2%), followed by Newar (3.5%), Brahmin (3.5%), and Tharu (3.2%). We observed no significant difference in the percentage of POAG among Brahmin, Newar, and Tharu (*p* > 0.05). The odds of Gurung having POAG were 2.05 times higher than for Brahmin, which was statistically significant (OR 2.05, 95% CI: 1.30–3.24).

### Association of hypertension or/and diabetes with POAG

Tables 2 and 3 are fourfold tables containing concordant and discordant pairs of cases and controls (POAG and without POAG) with and without exposure (to hypertension or diabetes) in all caste groups.

**Table 2:** Ethnicity-wise distribution of hypertension in cases and controls

		Without POAG (control)			
				
Ethnic group	With POAG (case)	Hypertensive	Non-hypertensive	Total	Odds ratio (95% CI)*
Newar	Hypertensive	17	37	54	2.06 (1.14–3.85)
	Non-hypertensive	18	141	159	
	Total	35	178	213	
Gurung	Hypertensive	10	33	43	3.66 (1.71–8.71)
	Non-hypertensive	9	117	126	
	Total	19	150	169	
Brahmin	Hypertensive	9	39	48	2.60 (1.40–5.07)
	Non-hypertensive	15	127	142	
	Total	24	166	190	
Tharu	Hypertensive	6	22	28	3.66 (1.44–11.05)
	Non-hypertensive	6	77	83	
	Total	12	99	111	
Total	Hypertensive	42	131	173	2.72 (1.95–3.88)
	Non-hypertensive	48	462	510	
	Total	90	593	683	

POAG: primary open-angle glaucoma; CI: confidence interval.

* Odds ratios were computed from discordant pairs.

**Table 3:** Ethnicity-wise distribution of diabetes in cases and controls

		Without POAG (control)			
				
Ethnic group	POAG (case)	Diabetic	Non-diabetic	Total	Odds ratio (95% CI)*
Newar	Diabetic	19	35	54	3.18 (1.58–6.95)
	Non-diabetic	11	148	159	
	Total	30	183	213	
Gurung	Diabetic	12	31	43	3.55 (1.65–8.46)
	Non-diabetic	8	118	126	
	Total	20	149	169	
Brahmin	Diabetic	16	32	48	2.66 (1.33–5.68)
	Non-diabetic	12	130	142	
	Total	28	162	190	
Tharu	Diabetic	7	21	28	2.15 (1.26–3.67)
	Non-diabetic	6	77	83	
	Total	13	98	111	
Total	Diabetic	53	120	173	3.50 (1.36–10.59)
	Non-diabetic	38	472	510	
	Total	91	592	683	

* Odds ratios were computed from discordant pairs.

Among Newar, we found 158 concordant pairs, of which 17 POAG pairs had hypertension and 141 pairs had no exposure to hypertension or POAG. Also among Newar, we found 55 discordant pairs – 18 pairs without POAG who had been exposed to hypertension, and 37 pairs with POAG who were not exposed to hypertension. The data for Gurung, Brahmin, and Tharu were similar (Table 2). In total, 42 concordant pairs had both POAG and hypertension, and 462 concordant pairs had neither. Among 179 discordant pairs, 48 pairs of controls (without POAG) had hypertension, and 131 pairs with POAG were non-hypertensive (Table 2).

Regarding diabetes, in total, we found 525 concordant pairs: 53 pairs had both POAG and diabetes, and 472 pairs had neither. Similarly, we found 38 discordant pairs without POAG but with diabetes and 120 discordant pairs with POAG but without diabetes (Table 3).

Additionally, we determined that hypertension and diabetes associated positively with POAG in each ethnic group (OR >1). The overall odds of having POAG increased 2.72-fold in patients with hypertension (Table 2) and 3.50-fold in patients with diabetes (Table 3).

### Visual acuity at the time of examination

We classified vision according to definitions by the International Council of Ophthalmology (26); 85.5% of POAG cases and 98.2% of controls had mild visual impairment or normal vision. Among POAG cases, 6.9% had moderate visual impairment, 2.9% had severe visual impairment, and 4.7% were blind. In contrast, only 1.8% of controls had moderate visual impairment, and none had severe visual impairment or was blind (Table 4).

**Table 4:** Vision at the time of presentation (cases versus controls)

Vision range*	Cases (*N*=173)	%	Controls (*N=*510)	%
6/6–6/18 (Mild visual impairment or normal vision)	148	85.5	501	98.2
6/24–6/60 (Moderate visual impairment)	12	6.9	9	1.8
5/60–3/60 (Severe visual impairment)	5	2.9	0	0
2/60-NPL or visual field <10° (Legal Blind)	8	4.7	0	0
Total	173	100	510	100

NPL: no perception of light.

* Vision based on the best corrected visual acuity of the better eye at the time of presentation.

## Discussion

### Demographic profile

Among all adults attending the hospital for eye consultation (*n* = 4,463), 183 (4.1%) were diagnosed with POAG for the first time, and 173 fulfilled the criteria for inclusion in the study. Although the number of males and females seeking eye care at the hospitals during the study period was almost equal, the frequency of POAG was 2.5-fold higher in males than females, concurring with an earlier study demonstrating that POAG occurs more commonly in males than females (28). The mean age for POAG participants in our study was 58.9±14.7 years. Similar to an earlier report (28), POAG incidence increased with age, but it declined in participants older than 75 years. Other investigators have demonstrated this pattern of decline after 70–75 years (29, 30). In the present study, the lower rate of POAG in this age group might have been due to the low average life expectancy in Nepal (31).

Based on the total number of individuals of each ethnic group attending the hospital, Gurung were found to have POAG more frequently (6.2%) than the rest. An earlier report by Rudnicka and colleagues reported variations in POAG prevalence among different ethnic groups like in this study (28).

### Association of POAG with hypertension and diabetes

Computed from discordant case–control pairs, the overall OR for our study population was 2.72, suggesting a stronger association between hypertension and POAG in Nepal and concurring with the results of the Blue Mountains Eye Study (10) and Rotterdam Study (32). The odds of a positive association between hypertension and POAG were higher among Gurung and Tharu (OR 3.66) compared with Newar (OR 2.06) and Brahmin (OR 2.60).

We also demonstrated a strong association between POAG and diabetes (OR 3.15), which concurs with a report that demonstrated a similar association between POAG and diabetes (33, 34). Our results suggest that POAG associates positively with hypertension and diabetes in all ethnic groups.

### Visual acuity at the time of presentation

Eighty-five percent of POAG participants had normal to mild impaired vision in their better eye compared with normal vision in 98% of controls, possibly due to the opportunistic screening for POAG that we conducted for this study. Thus, most cases could have been in the early stage of the disease.

However, 9.8% of POAG cases had impaired vision of varying degree, and 4.7% were blind when diagnosed. In contrast, the controls showed no blindness. Compared to Kooner et al. (34), our study showed a lower rate of blindness (21.7%) in POAG cases at the time of diagnosis. This discrepancy is largely due to differing definitions of blindness. Kooner defined blindness as vision equal to 6/60 or less and/or a visual field less than 20°, whereas our definition considered blindness as visual acuity less than 3/60 and/or a visual field less than 10°. Although the percentage of blindness was only 4.7% in this study, the overall impact could be greater than what we perceive at this point.

According to the latest demographic profile of Nepal from July 2013 (35), the country has a population of over 29.8 million. Of these, 66.5% (19.8 million) belong to the age group of 15 years or older. Even if one is considering the lowest POAG prevalence to make a nationwide estimate, such as 3.25% for the Tharu ethnic group in our study, this would tentatively suggest 645,585 people with POAG in Nepal. Based on our findings that 4.7% people were blind at the time of first diagnosis, it can be additionally estimated that 30,342 people could be blind in Nepal due to POAG. The impact is likely larger, as the odds of developing POAG are higher in diabetic (OR 3.50) and hypertensive (OR 2.72) patients.

In conclusion, our study reports for the first time a significant association between POAG, hypertension, and diabetes in a representative sample of the Nepalese population. The therapeutic implications of this finding suggest that ophthalmologists and other eye care practitioners should not only examine their patients for effects of hypertensive and diabetic retinopathies, but also screen them for overt or occult signs of glaucoma by simple fundus examination and measurement of intraocular pressure. If necessary, patients should be referred to the higher center with specialized glaucoma services for detailed glaucoma evaluation. Conversely, general practitioners, internists, cardiologists, and endocrinologists should recommend glaucoma screening for patients with diabetes or hypertension.
